# The Percentage of Free PSA and Urinary Markers Distinguish Prostate Cancer from Benign Hyperplasia and Contribute to a More Accurate Indication for Prostate Biopsy

**DOI:** 10.3390/biomedicines8060173

**Published:** 2020-06-25

**Authors:** Zlata Huskova, Jana Knillova, Zdenek Kolar, Jana Vrbkova, Milan Kral, Jan Bouchal

**Affiliations:** 1 Department of Clinical and Molecular Pathology, Faculty of Medicine and Dentistry, Palacky University and University Hospital, 779 00 Olomouc, Czech Republic; zlata.huskova@upol.cz (Z.H.); jana.knillova@upol.cz (J.K.); zdenek.kolar@upol.cz (Z.K.); 2 Institute of Molecular and Translational Medicine, Faculty of Medicine and Dentistry, Palacky University, 779 00 Olomouc, Czech Republic; jana.vrbkova@upol.cz; 3 Department of Urology, University Hospital, 779 00 Olomouc, Czech Republic

**Keywords:** prostate cancer, % free PSA, *PCA3*, *AMACR*, inflammation

## Abstract

The main advantage of urinary biomarkers is their noninvasive character and the ability to detect multifocal prostate cancer (CaP). We have previously implemented a quadruplex assay of urinary markers into clinical practice (*PCA3, AMACR, TRPM8* and *MSMB* with *KLK3* normalization). In this study, we aimed to validate it in a larger cohort with serum PSA 2.5–10 ng/mL and test other selected transcripts and clinical parameters, including the percentage of free prostate-specific antigen (PSA) (% free PSA) and inflammation. In the main cohort of 299 men, we tested the quadruplex transcripts. In a subset of 146 men, we analyzed additional transcripts (*CD45, EPCAM, EZH2, Ki67, PA2G4, PSGR, RHOA* and *TBP*). After a prostate massage, the urine was collected, RNA isolated from a cell sediment and qRT-PCR performed. Ct values of *KLK3* (i.e., PSA) were strongly correlated with Ct values of other genes which play a role in CaP (i.e., *PCA3, AMACR, TRPM8, MSMB* and *PSGR*). *AMACR, PCA3, TRPM8* and *EZH2* mRNA expression, as well as % free PSA, were significantly different for BPH and CaP. The best combined model (% free PSA plus *PCA3* and *AMACR*) achieved an AUC of 0.728 in the main cohort. In the subset of patients, the best AUC 0.753 was achieved for the combination of *PCA3*, % free PSA, *EPCAM* and *PSGR*. *PCA3* mRNA was increased in patients with inflammation, however, this did not affect the stratification of patients indicated for prostate biopsy. In conclusion, the percentage of free PSA and urinary markers contribute to a more accurate indication for prostate biopsy.

## 1. Introduction

The definitive diagnosis of prostate cancer (CaP) is based on the results of a prostate biopsy. Due to the complications associated with prostate biopsy, including hospitalization, bleeding, sepsis and pain, it is important to reduce the number of unnecessary biopsies done. For almost 30 years, the decision to biopsy a patient has relied on elevated prostate-specific antigen (PSA) levels or abnormal findings on a digital rectal exam (DRE). Serum PSA is also included in seven well-known multivariable risk calculators which may be used both by patients and clinicians [1]. Besides serum markers, urinary biomarkers may also help urologists to make decisions concerning diagnosis and prognosis. Both serum and urine markers have been recently thoroughly reviewed [2,3,4]. In addition to urine tests and the blood Prostate Health Index or 4KScore, magnetic resonance imaging (MRI) is also an important option [5,6,7,8,9,10]. Novel approaches are being tested too, e.g., DNA methylation assays, miRNAs and lncRNAs [11,12,13].

Based on a preliminary analysis of a subset of patients, we have reported that prostatic inflammation may affect urine *PCA3* mRNA levels [14]. There is only one study which analyzed a subgroup of patients with serum PSA 4–10 ng/mL [15], however, the group of BPH (benign prostate hyperplasia) without inflammation was rather small. On the other hand, a well-defined case study supports a potential upregulation of *PCA3* score due to inflammation [16].

Most commonly used, the Progensa urine assay has been approved by FDA and *KLK3* (i.e., PSA) mRNA levels are important for calculation of the PCA3 score (*PCA3* mRNA/*PSA* mRNA × 1000). Similar normalization to the prostate specific *KLK3* levels is also used in PCR-based assays which enable analysis of additional transcripts of interest. Slightly elevated *KLK3* mRNA levels had been reported in urine samples from patients with CaP [17] and therefore we also assayed *TBP* (TATA-binding protein) and *RHOA* (Ras homolog gene family member A) as potential alternative house-keeping genes in the current study. Previously, we have successfully analyzed *PCA3* in combination with *AMACR* (a-methylacyl-CoA racemase), *EZH2* (enhancer of zeste homolog 2), *MSMB* (microseminoprotein, β) and *TRPM8* (transient receptor potential cation channel, subfamily M member 8) [18]. In the current study, we were interested if additional markers can improve the stratification of BPH and CaP patients. *EPCAM* (epithelial cell adhesion molecule) is frequently used as a marker of circulating tumor cells [19,20] but it has not been evaluated in patients’ urine yet. There are also no urine-based studies for *Ki67* (*MKI67*, marker of proliferation *Ki67*). Two studies have reported promising results for cancer detection using *PSGR* (prostate-specific G-protein coupled receptor, official gene name *OR51E2*, olfactory receptor *51E2*) [17,21]. We have also included *PA2G4* which acts as an androgen receptor corepressor and may be downregulated in prostate cancer [22]. Last but not least, we were interested if prostate inflammation is reflected by a higher incidence of leukocytes and their specific marker *CD45* in the urine.

In this study, we aimed to validate and improve our established quadruplex urine test ([18]; *AMACR, PCA3, MSMB, TRPM8, EZH2* with *KLK3* normalization) in a cohort of patients with serum PSA 2.5–10 ng/mL. The percentage of free PSA in serum, inflammation in benign prostate tissue and panel of candidate urine transcripts were also evaluated.

## 2. Material and Methods

### 2.1. Patients, Inflammation, Urine Collection and RNA Isolation

**Figure 1:**
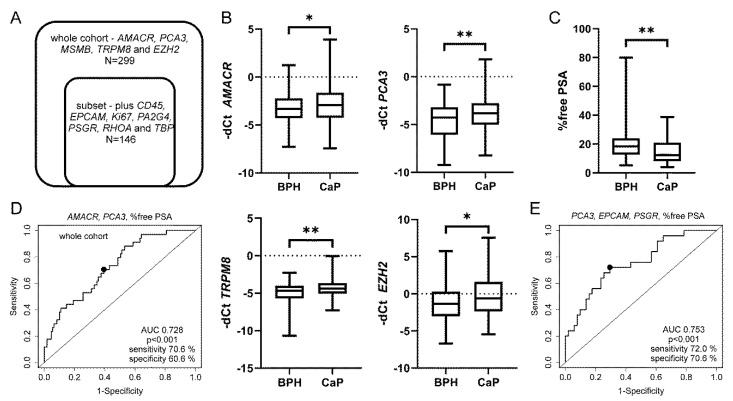
Urine markers are differentially expressed between CaP and BPH. (**A**) Description of the whole cohort and a subset of patients analyzed for indicated transcripts. (**B**) *AMACR, PCA3*, *TRPM8 and EZH2* expression were significantly different for CaP and BPH patients (*p* values 0.045, 0.004, 0.005 and 0.019, respectively). (**C**) Percentage of free PSA in combination with urine markers contribute to discrimination of BPH from CaP. Percentage of free PSA significantly discriminates CaP from BPH (*p* value 0.003). Box-plots represent median, 25–75% percentiles and range of values. *p*-values <0.05 and <0.01 are indicated by * and **, respectively. (**D**) Receiver-operating characteristic analysis for % free PSA in combination with urinary markers *AMACR* and *PCA3* or (**E**) with *EPCAM, PCA3* and *PSGR* in the subset of patients.

The patient clinical and pathological characteristics are shown in Table 1 and Figure 1A. The study was approved by the Ethics Committee of the University Hospital Olomouc and Medical Faculty of Palacky University in June 2014 (Ref No. 127/14). Verbal informed consent was obtained from all individual participants for the measurement of urine markers as a part of their standard urological examination and for the anonymous data analysis in this study. Written informed consent would have been redundant as the routine clinical procedure was not affected by the noninvasive measurement of urine markers at all. Patients with BPH were divided into two subgroups, those with and those without extensive inflammation according to the histopathological examination by an experienced pathologist (ZK). Urine (30 mL) was collected after a massage of the prostate during the digital rectal examination and processed within 1 h from the collection. The urine samples were centrifuged at 650× *g* (5 min at 4 °C) and the cell sediment was used for total RNA isolation by SurePrep Urine Exfoliated Cell RNA Purification Kit (Fisher Scientific). The concentration of RNA was measured on Nanodrop. RNA samples were stored at −80 °C.

### 2.2. Reverse Transcription and Quantitative Polymerase Chain Reaction

Total RNA (100 ng) was treated with DNase I (Invitrogen) and then transcribed to cDNA with SuperScript III Reverse Transcriptase (Invitrogen). The quantitative real time polymerase chain reaction (qRT-PCR) was performed with LightCycler 480 Probes Master Mix (Roche) and appropriate primers (Table S1) for 50 cycles of denaturation, annealing and extension (95–60–72 °C each for 20 s) on LightCycler 480 instrument (Roche). All samples were analyzed in duplicate. Samples with *KLK3* Ct values >35 (Ct, cycle threshold) and missing values for two or more urine markers were excluded due to a low amount of cDNA. Relative quantification was carried out according to the delta Ct (dCt) method using a reference gene (dCt = Ct_target_ − Ct*_KLK3_*) and inverse values (−dCt) were used for subsequent statistical analysis and visualization.

### 2.3. Dilution of LNCaP Cells and Leukocytes

Prostate cancer cells (LNCaP) were harvested from a standard cell culture and counted in a hemocytometer. Leukocytes were obtained from 5 mL of EDTA-stabilized blood from a healthy volunteer. After centrifugation at 2300× *g*, buffy coat was aspirated and lysis of residual erythrocytes was performed with PBS and ddH2O and centrifuged for 5 min at 300 g. Leukocytes were counted in a hemocytometer. Dilution of prostate cancer LNCaP cells and leukocytes was performed according to Figure S2B,C and processed as standard urine samples.

### 2.4. Statistical Analysis

The data were analyzed with respect to the clinical-pathological parameters (percentage of free PSA, inflammation in benign tissue, Gleason score and tumor stage) using the program Statistica 12 (TIBCO Software Inc., Palo Alto, CA, USA, Mann–Whitney test, Spearman’s rank correlation coefficient and Pearson´s chi-squared test). Graphs were generated in GraphPad Prism 8 (GraphPad Software, San Diego, CA, USA). Univariate and multivariate logistic regression analyses were performed with software R, ver. 3.5.0 (www.r-project.org). The best multivariate model was always chosen from a group of models generated in best subset selection process (using the bess function from BeSS R package, ver. 1.0.5) as a model with the smallest testing error rate estimated by LOOCV. ROC curves were generated with function ROC from package Epi, ver. 2.30, while AUCs (areas under the ROC curve) altogether with 95% confidence intervals (computed with 2000 stratified bootstrap replicates) were calculated with functions from pROC package, ver. 1.12.1. The *p*-values which address the null hypothesis that the AUC is 0.5 were calculated using the Wilcox test function using the roc.area function from verification package, ver. 1.42.

## 3. Results

### 3.1. Urine KLK3 Strongly Correlates with Prostate Cancer Relevant Transcripts

The patients clinical and pathological characteristics are shown in Table 1. Our expression analysis of 13 urine transcripts enabled a unique analysis using a non-parametric Spearman’s rank correlation coefficient (Table 2). As expected, *KLK3* Ct values strongly correlated with Ct values of other genes which have been reported to play a role in prostate cancer (i.e., *PCA3, AMACR, TRPM8, MSMB* and *PSGR*). A weaker correlation was observed for *EPCAM* which suggests the presence of other epithelial cells from the urinary tract. *CD45* expression strongly correlated with *EZH2* and in particular with house-keeping genes *TBP* and *RHOA*, indicating the abundant presence of leukocytes in urine.

As elevated *KLK3* mRNA levels had been reported in urine samples from patients with CaP [17], we also assayed other house-keeping genes. However, none of the genes used for alternate normalization (*TBP, RHOA, PA2G4, EPCAM*) outperformed *KLK3*. In fact, there was only a trend for decreased *KLK3* in CaP compared to BPH when normalized to *EPCAM* (*p* = 0.083) and a trend for increased *PCA3* in CaP when normalized to *PA2G4* (*p* = 0.095) in the relevant subset of patients (Figure 1A). Therefore standard normalization to *KLK3* Ct values was used for all urine markers below.

### 3.2. Urine PCA3, AMACR and Percentage of Free PSA Discriminate CaP from BPH

The major aim of this study was validation of our previous results (*AMACR, PCA3, MSMB, TRPM8, EZH2* with *KLK3* normalization) in an extended cohort of patients with total PSA levels 2.5–10 ng/mL (Table 1). A subset of patients (*n* = 146) was also evaluated for additional transcripts (*CD45, EPCAM, Ki67, PA2G4, PSGR, RHOA, TBP*, Figure 1A, please see below Section 2.3). In the whole cohort benign cases with PSA levels above 10 ng/mL (*n* = 31) and cancer cases below 2.5 ng/mL (*n* = 10) were also included (Table 1). Significantly different expressions between BPH and CaP were found for *AMACR* (*p* = 0.045), *PCA3* (*p* = 0.004), *TRPM8* (*p* = 0.005) and *EZH2* (*p* = 0.019, Figure 1B). As expected, % of free PSA was also significantly different for BPH and CaP cases (*p* value 0.003, Figure 1C). Importantly, the percentage of free PSA alone had an AUC 0.66 (Table 3). Multivariate logistic analysis of all parameters identified the best combined model (% free PSA plus *PCA3* and *AMACR*) with improved AUC 0.728 (Table 3, Figure 1D).

### 3.3. The Best Combined Model was Achieved for EPCAM, PSGR, PCA3 and Percentage of Free PSA in the Subset of Patients

We were also interested if novel markers (*EPCAM, PSGR, Ki67, PA2G4*) can improve stratification of BPH and CaP in a subset of patients (*n* = 146, Figure 1A). Other aims were to evaluate the normalization of gene expression (*KLK3, RHOA* and *TBP*; please see above Section 2.1) and to analyze the presence of leukocytes in the urine (*CD45*; please see Section 2.4 below). Although none of the novel transcripts were different between BPH and CaP, the multivariable analysis revealed an alternative combination of *EPCAM, PSGR, PCA3* and % free PSA with AUC 0.753 (Table 3, Figure 1E). However, this promising model needs to be validated on a larger cohort of patients.

### 3.4. Prostate Inflammation in BPH Increases Urine PCA3 but Does Not Affect Prostate Biopsy Decision Making

**Figure 2:**
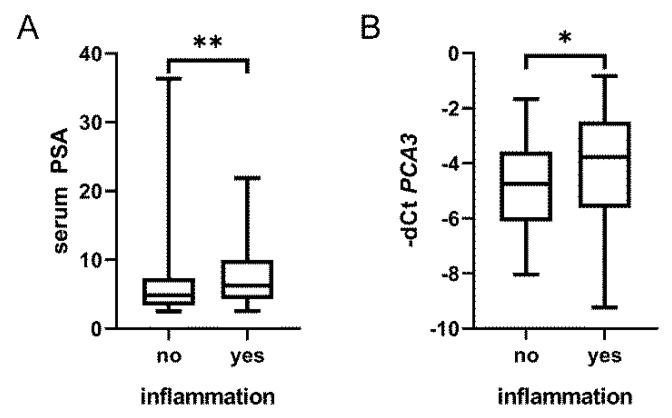
Inflammation in BPH tissue affects serum PSA and urine *PCA3*. Serum PSA levels (**A**) and to a lesser extent also urine *PCA3* (**B**) were significantly higher in patients with inflamed BPH tissue (*p* values 0.005 and 0.040, respectively). Box-plots represent median, 25–75% percentiles and range of values. *p*-values <0.05 and <0.01 are indicated by * and **, respectively.

One of the first studies on *PCA3* by Hessels et al. provided data on inflammation in 84 patients with no malignancy [23]. There was no association of inflammation with *PCA3*, and surprisingly, also not with serum PSA (Figure S1). In our previous study [24], we histopathologically evaluated inflammation in needle biopsies of BPH. Based on a preliminary analysis, we reported that prostatic inflammation may affect urine *PCA3* [13]. This observation was confirmed in the present study where both *PCA3* (*p* = 0.041) and serum PSA (*p* = 0.005) were significantly increased in patients with inflammation (Figure 2). Importantly, the slight increase of *PCA3* did not significantly alter stratification of BPH cases with and without inflammation into risk groups based on the combined evaluation of *PCA3, AMACR* and % free PSA (*p* = 0.89).

### 3.5. The qRT-PCR Assay Detects Relevant Numbers of Leukocytes and Cancer Cells in Urine

As mentioned above, *CD45* mRNA was abundant in urine samples (Table 2, Figure S2A), but the levels were no different in benign patients with and without inflammation (*p* = 0.17). We were interested if our qRT-PCR assay detects relevant numbers of leukocytes (*CD45* positive) and cancer cells (*KLK3* positive) in urine and performed a simple dilution experiment (see Methods 4.3). As reported by others, 30 mL of urine may contain 100,000 leukocytes which may further increase during urinary tract inflammation [25,26,27]. This number of leukocytes from a volunteer provided a Ct value of *CD45* close to 30 (Figure S2B) which was very similar to the mean of our urine samples (*CD45* mean 29.6). The mean Ct value for *KLK3* from patient samples was 31.2 which was close to the value of 1000 LNCaP prostate cancer cells (Figure S2C). These results are in good concordance with an image-based analysis which consistently detected 1000 LNCaP cells spiked in urine [19]. Tens to several thousands of cancer cells were detected in urine samples from 35 patients [19] which is well mirrored in the variability of Ct values from our patients´ samples (Figure S2A).

## 4. Discussion

Our study supports a combined analysis of urine and blood tests for the more accurate indication for prostate biopsy. Best results were achieved with a combination of *AMACR, PCA3* and % free PSA. A separate analysis of *PCA3* and % free PSA has previously been reported [28,29]. For example, Ploussard et al. found PCA3 score superior to % free PSA in patients with “grey zone” PSA values (2.5–10 ng/mL) [28]. Auprich et al. examined patients at first, second, and ≥third repeat biopsy sessions [29]. At first repeat biopsy, *PCA3* predicted CaP best while % free PSA performed best at second and ≥third repeat biopsies. Clinicians and patients have many other options for refinement of prostate biopsy decision making, depending on available methods and funding. Besides the urine Select MDx, ExoDx and *TMPRSS2-ERG* [30,31,32,33,34,35] and the blood Prostate Health Index and 4KScore [5,6,7,8], MRI is also an important option [9]. Combination of multiparametric MRI (mp-MRI) with urinary *PCA3* or Select MDx has recently shown a potential to reduce unnecessary biopsies, which could, in turn, prevent overdiagnosis and overtreatment [10,32]. Multiparametric MRI has recently been established in our hospital and we also aim to implement it in the prospective validation of our models. In particular, the value of *EPCAM* and *PSGR* urinary transcripts deserves further analysis as they have so far been reported only in few studies [17,21,36].

The validity of our method was reflected in the strong correlation of prostate related genes, i.e., *KLK3, PCA3, AMACR, TRPM8, MSMB* and *PSGR*. A weaker correlation was observed for *EPCAM* which suggests the presence of other epithelial cells from the urinary tract. Still, combined evaluation of *EPCAM, PSGR, PCA3* and percentage of free PSA showed promising results in the discrimination of CaP and BPH patients. We have not proved the importance of *PA2G4* and *Ki67* for CaP detection as they weakly correlated with some prostate cancer-related markers (e.g., *AMACR*), but also with leukocyte-specific *CD45* and house-keeping genes (*TBP* and *RHOA*). *CD45* expression strongly correlated with *EZH2* and in particular with house-keeping genes *TBP* and *RHOA*, indicating the abundant presence of leukocytes in urine.

*CD45* mRNA was abundant in urine samples but the levels were no different in benign patients with and without inflammation. However, we only had information on inflammation from the prostate tissue which is probably a minor source of leukocytes in the urine. Based on a preliminary analysis, we have previously reported that prostatic inflammation may affect urine *PCA3* [14]. This observation was confirmed in the current study where both *PCA3* (*p* = 0.041) and serum PSA (*p* = 0.005) were increased in patients with inflammation. This is in line with a case report where symptomatic male urethral *Chlamydia trachomatis* infection resulted in inflammation of the prostate, with associated increases in both PSA and *PCA3* [16]. Although several articles have reported no association between *PCA3* and inflammation, most of these included patients with any serum PSA level. One of the first studies analyzed *PCA3* in 84 patients with no malignancy and with no limit on serum PSA levels [23]. From the data provided, there was no association of inflammation with *PCA3*, but surprisingly, also not with serum PSA. No association between *PCA3* and inflammation was also reported in other cohorts with no limit on serum PSA [37,38,39]. The third study [39] consisted of young patients and provided no information on serum PSA, which was also commented on elsewhere [40]. The last study on *PCA3* and inflammation analyzed a subgroup of patients with serum PSA levels 4–10 ng/mL [15]. *PCA3* was no different for patients with prostatitis and patients with BPH. However, the group with BPH without inflammation was rather small (30 patients with any serum PSA level and the exact number of the patients with “grey zone” PSA values was not specified). Although we found a slight increase of *PCA3* in patients with inflammation, this did not affect the stratification of patients indicated for prostate biopsy. Last but not least, prostate inflammation and its evaluation in urine will deserve further research by different methods, such as urine test strips, image-based analysis or mass spectrometry.

In summary, our study supports a combined analysis of urine and blood tests for the more accurate indication for prostate biopsy. Best results were achieved with a combination *of AMACR, PCA3* and % free PSA. *PCA3* was slightly increased by tissue inflammation but without effect on the interpretation of the urine test.

## Figures and Tables

**Table 1:** Patients characteristics.

Characteristics	Whole Cohort (*n* = 299)		Subset (*n* = 146)	
	BPH	CaP	BPH	CaP
No. Patients	178	121	78	68
Age ^1^				
<55	17	18	7	7
55–65	80	46	31	26
>65	81	57	40	35
Serum PSA (ng/mL)				
<2.5	-	10	-	6
2.5–10	147	111	63	62
>10	31	-	15	-
% free PSA ^2^				
0–10	18	12	11	9
11–20	49	16	20	12
21–100	48	8	24	5
Inflammation ^3^				
yes	62	-	34	-
no	83	-	43	-
n.a.	33	-	1	-
Gleason score				
<7	-	39	-	18
7	-	70	-	41
>7	-	12	-	9
Cancer stages ^4^				
T1-pT2b	-	38	-	18
pT2c	-	64	-	39
pT3a-b	-	19	-	11
Risk groups ^5^				
low	-	26	-	10
intermediate	-	70	-	42
high	-	25	-	16

^1^ There is no difference in age between BPH and CaP in any cohort (*p* values 0.89 and 0.93, respectively). ^2^ Measurement of % free PSA was not available for all patients. ^3^ Inflammation was considered in BPH tissue only. ^4^ No patient was diagnosed with lymph node or distant metastases. ^5^ Risk groups were defined as low (T1-pT2b and Gleason score < 7), intermediate (pT2c or Gleason score 7) and high (pT3 or Gleason score > 7). No patients with a particular parameter are indicated by “-”.

**Table 2:** Spearman´s correlations between Ct values with emphasis on *KLK3* and *CD45*.

	*KLK3*	*AMACR*	*PCA3*	*TRPM8*	*MSMB*	*EZH2*	*CD45*	*EPCAM*	*Ki67*	*RHOA*	*PA2G4*	*PSGR*	*TBP*
*KLK3*	1.000	**0.720**	**0.791**	**0.863**	**0.895**	0.292	*−0.092*	0.489	*0.154*	*0.192*	0.433	**0.826**	*0.169*
*AMACR*		1.000	0.685	0.663	0.680	0.562	*0.186*	0.507	0.350	0.463	0.665	0.647	0.431
*PCA3*			1.000	**0.770**	0.682	0.274	*−0.027*	0.426	*0.191*	*0.240*	0.411	**0.747**	*0.225*
*TRPM8*				1.000	**0.827**	0.317	*0.007*	0.582	*0.256*	0.324	0.506	**0.746**	0.318
*MSMB*					1.000	0.403	*0.050*	0.462	*0.184*	*0.266*	0.474	**0.711**	*0.215*
*EZH2*						1.000	**0.775**	0.259	0.530	**0.738**	0.669	*0.039*	0.685
*CD45*							1.000	0.157	0.491	**0.809**	0.588	*−0.126*	**0.718**
*EPCAM*								1.000	0.500	0.425	0.496	0.368	0.457
*Ki67*									1.000	0.586	0.624	*0.075*	0.626
*RHOA*										1.000	**0.819**	*0.105*	**0.926**
*PA2G4*											1.000	0.384	**0.827**
*PSGR*												1.000	*0.126*
*TBP*													1.000

Strong correlations (Rs > 0.7) are highlighted in bold. Insignificant results (*p* > 0.001) are in italics.

**Table 3:** Univariate and multivariate analysis.

**Univariate Logistic Analysis**				**ROC Analysis**	
**Variable**	**β**	**OR (95% CI)**	***p*-Value**	**AUC (95% CI)**	***p*-Value**
*AMACR*	0.168	1.183 (1.031–1.357)	0.017	0.569 (0.502–0.635)	0.023
*PCA3*	0.233	1.263 (1.09–1.463)	0.002	0.602 (0.533–0.667)	0.002
*TRPM8*	0.341	1.406 (1.143–1.73)	0.001	0.599 (0.532–0.664)	0.003
*MSMB*	0.07	1.072 (0.883–1.303)	0.482	0.526 (0.458–0.594)	0.223
*EZH2*	0.127	1.136 (1.037–1.244)	0.006	0.587 (0.519–0.653)	0.007
*EPCAM*	0.141	1.152 (0.991–1.338)	0.065	0.585 (0.488–0.674)	0.042
*PSGR*	0.127	1.135 (0.888–1.451)	0.311	0.532 (0.44–0.633)	0.255
% free PSA	−0.075	0.928 (0.879–0.98)	0.007	0.666 (0.559−0.763)	0.001
**Multivariate Logistic Analysis**				**ROC Analysis**	
**Whole Cohort**	**β**	**OR (95% CI)**	***p*-Value**	**AUC (95% CI)**	***p*-Value**
age	0.043	1.044 (0.97–1.123)	0.256	0.728 (0.633–0.816)	<0.001
*AMACR*	0.244	1.277 (0.943–1.729)	0.114		
*PCA3*	0.283	1.327 (1.01–1.742)	0.042		
% free PSA	−0.082	0.921 (0.871–0.975)	0.004		
**Subset**	**β**	**OR (95% CI)**	***p*-Value**	**AUC (95% CI)**	***p*-Value**
*PCA3*	0.439	1.551 (1.092–2.205)	0.014	0.753 (0.642–0.862)	<0.001
*EPCAM*	0.207	1.23 (0.935–1.619)	0.139		
*PSGR*	−0.151	0.86 (0.578–1.278)	0.454		
% free PSA	−0.064	0.938 (0.877–1.004)	0.064		

AUC, area under curve; OR, odds ratio; ROC, receiver-operating characteristic; β, beta coefficient; 95% CI, 95% confidence interval.

## Supplementary Materials

The following are available online at https://www.mdpi.com/2227-9059/8/6/173/s1.
