# Supplementary material for: The Percentage of Free PSA and Urinary Markers Distinguish Prostate Cancer from Benign Hyperplasia and Contribute to a More Accurate Indication for Prostate Biopsy
Source: Biomedicines. 2020 Jun 25;8(6):173. doi: 10.3390/biomedicines8060173 (PMC7344460; doi:10.3390/biomedicines8060173)
Supplement: Supplementary file 1 [file biomedicines-08-00173-s001.pdf]

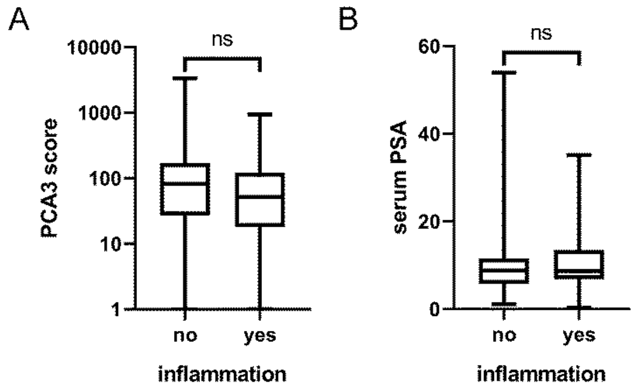

Figure S1 . PCA3 score (**A**), but also serum PSA (**B**) were not affected by inflammation in the study by Hessels *et al.* [23] (p=0.226 and p=0.655, respectively; ns, not significant; box-plots were generated from data provided in the original article).

A

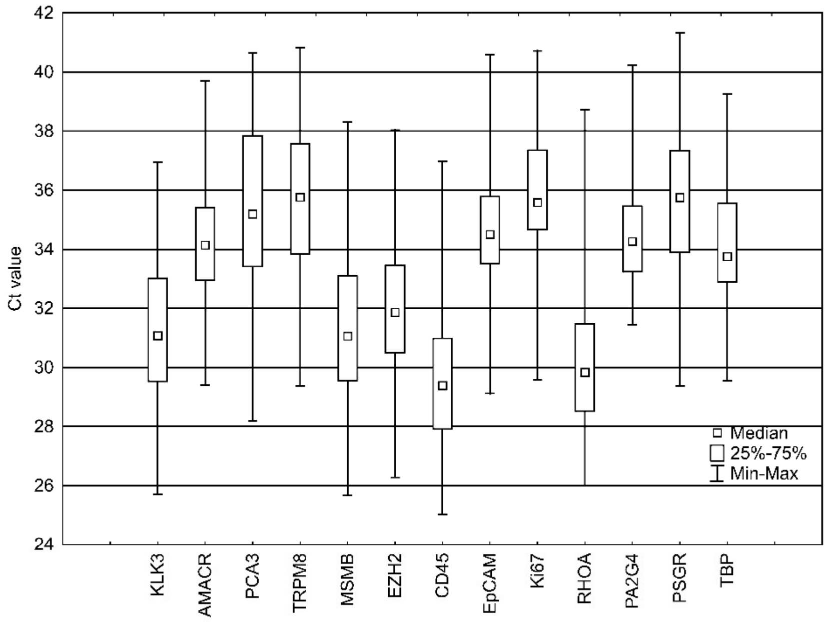

B

LNCaP	Leukocytes	log leu
100 000	10	1
100 000	100	2
100 000	1000	3
90 000	10 000	4
1000	100 000	5

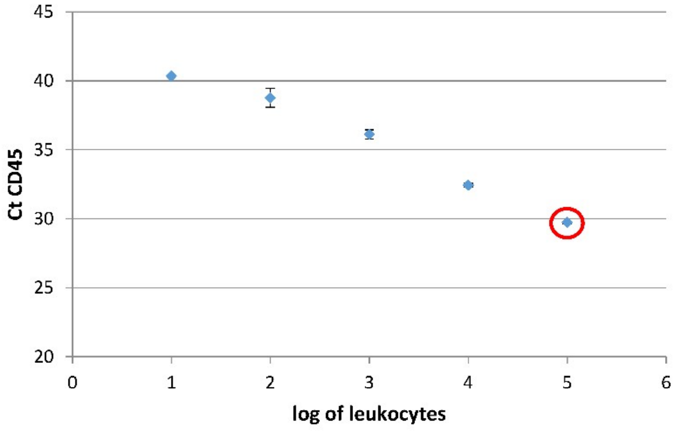

C

LNCaP	Leukocytes	log LNCaP
10	100 000	1
100	100 000	2
1000	100 000	3
10 000	90 000	4
100 000	1000	5

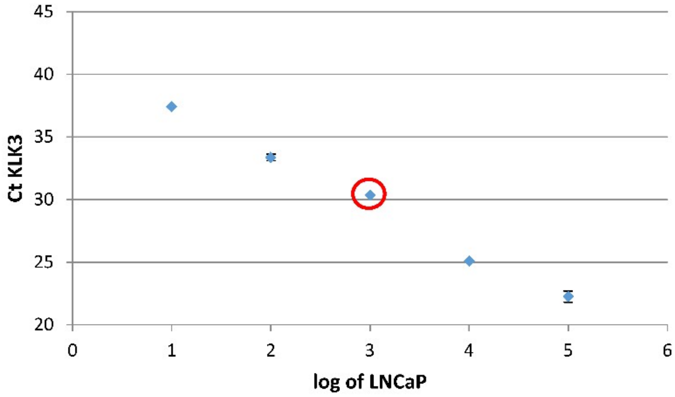

Figure S2 . Results of dilution experiments with leukocytes and LNCaP prostate cancer cells are in good concordance with real Ct values from patients samples. (**A**) Range of Ct values of the urine transcripts from all patients. Box-plots represent median, 25%-75% percentiles and range of values. (**B**) Dilution serie of leukocytes in prostate cancer LNCaP cells. One hundred thousands of leukocytes gave Ct value of CD45 close to 30 which was very similar to the mean from our urine samples (CD45 mean 29.6). (**C**) Dilution serie of LNCaP cells in leukocytes. Mean of Ct value for KLK3 from patients samples was 31.2 which was close to the value of 1000 LNCaP prostate cancer cells. Red cycle shows Ct values around 30.

Table S1. Primers and probes used in the study.

Marker[1]	Gene ID	Primers and probes	Position or cat.no.[2]	Product
AMACR	NM_001167595.1	FW-5′-TCAACTATTTGGCTTTGTCAGG-3′<br>RV-5′-GTGAGAATCCGTATGCCCC-3′<br>probe UPL #29	exon 2/3<br>exon 3	67 bp
CD45/PTPRC	NM_001267798.1	TaqMan Gene Expression Assay<br>probe FAM-MGB	Hs04189704_m1	57 bp
EPCAM	NM_002354.2	FW-5′-AGTTGGTGCACAAAATACTGTCAT-3′<br>RV-5′-TCCCAAGTTTTGAGCCATTC-3′<br>probe UPL #8	exon 2<br>exon 3	89 bp
EZH2	NM_004456.3	FW-5′-GATGATGGAGACGATCCTGAA-3′<br>RV-5′-GGGCGGCTTTCTTTATCAT-3′<br>probe UPL #65	exon 5<br>exon 5/6	80 bp
Ki67/MKI67	NM_002417.4	FW-5′-TCAAGGAACTGATTCAGGAGAAG-3′<br>RV-5′-GTGCACTGAAGAACACATTTCC-3′<br>probe UPL #32	exon 13<br>exon 13	77 bp
KLK3	NM_001648.2	FW-5′-GTCTGCGGCGGTGTTCTG-3′<br>RV-5′-GCCGACCCAGCAAGATC-3′<br>5′-FAM-CACAGCTGCCCACTGCATCAGGA-BHQ1-3′	exon 2<br>exon 3<br>exon 2	88 bp
MSMB	NM_002443.2	FW-5′-AAATTTCATGTTGCACCCTTG-3′<br>RV-5′-CCATTCACTGACAGAACAGGTC-3′<br>probe UPL #22	exon 3/4<br>exon 4	140 bp
PA2G4	NM_006191.2	TaqMan Gene Expression Assay<br>probe FAM-MGB	Hs00854538_g1	113 bp
PCA3	NR_015342.1	FW-5′-GCACATTTCCAGCCCCTTTAAA-3′<br>RV-5′-GGGCGAGGCTCATCGAT-3′<br>5′-FAM-AGAAATGCCCGGCCGCCATC-BHQ1-3′	exon 3<br>exon 4<br>exon 4	113 bp
PSGR/OR51E2	NM_030774.3	TaqMan Gene Expression Assay<br>probe FAM-MGB	Hs00258239_s1	74 bp
RHOA	NM_001313941.1	TaqMan Gene Expression Assay<br>probe FAM-MGB	Hs00357608_m1	62 bp
TBP	NM_003194.5	FW:5′-CACGAACCACGGCACTGATT-3′<br>RV:5′-TTTTCTTGCTGCCAGTCTGGAC-3′<br>5′-Cy5-TCTTCACTCTTGGCTCCTGTGCACA-BHQ2-3′	exon 5<br>exon 6<br>exon5/6	89 bp
TRPM8	NM_024080.4	FW-5′-TCAGAATAAGAAGGAACTCTCCAAA-3′<br>RV-5′-CAGCAGCATTGATGTCGTTC-3′<br>probe UPL #32	exon 13<br>exon 14	125 bp

[1] Gene names are as follows - alpha-methylacyl-CoA racemase (*AMACR*), leukocyte common antigen (*CD45*; official gene name *PTPRC*, Protein Tyrosine Phosphatase, Receptor Type C), epithelial cell adhesion molecule (*EPCAM*), enhancer of zeste homolog 2 (*EZH2*), marker of proliferation *Ki67* (*MKI67*), human kallikrein 3 (*KLK3*, also known as PSA, prostate specific antigen), beta-microseminoprotein (*MSMB*), proliferation associated protein 2G4 (*PA2G4*), prostate cancer antigen 3 (*PCA3*), prostate-specific G-protein coupled receptor (*PSGR*; official gene name *OR51E2*, olfactory receptor 51E2), Ras homolog gene family member A (*RHOA*), TATA-binding protein (*TBP*), transient receptor potential cation channel subfamily M member 8 (*TRPM8*).

[2] Indicated assays were purchased from Thermo Fisher Scientific.

Additional abbreviations are as follows: black hole quencher 1 (BHQ1), universal probe library (UPL), fluorescein amidite (FAM), minor groove binder (MGB)
